# Integrating Molecular Modeling and Nanoemulsion Characterization for Ibuprofen

**DOI:** 10.1021/acsomega.5c09579

**Published:** 2026-02-25

**Authors:** Antônio S. N. Aguiar, Luana A. F. Afiune, Vitória A. M. Silva, Rodrigo A. B. Lopes-Martins, Lucas D. Dias, Alberto S. S. Filho, James O. Fajemiroye, Leonardo L. Borges, Hamilton B. Napolitano

**Affiliations:** † Laboratório de Novos Materiais, Universidade Evangélica de Goiás, Anápolis 75083-515, Goiás, Brazil; ‡ Grupo de Química Teórica e Estrutural de Anápolis, 271384 Universidade Estadual de Goiás, Anápolis 75132-400, Goiás, Brazil; § Laboratório de Pesquisas em Biodiversidade, Universidade Evangélica de Goiás, Anápolis 75083-515, Goiás, Brazil; ∥ Centro Universitário UniRedentor, Itaperuna CEP 28300-000, Rio de Janeiro, Brazil; ⊥ Instituto de Ciências Biológicas, 67824 Universidade Federal de Goiás, Goiânia 74690-900, Goiás, Brazil; # Escola de Ciências Médicas e da Vida, 125092 Pontifícia Universidade Católica de Goiás, Goiânia 74605-010, Goiás, Brazil

## Abstract

Ibuprofen (IBU), a widely used nonsteroidal anti-inflammatory drug, exhibits low aqueous solubility and polymorphic behavior, which can compromise its bioavailability and pharmaceutical performance. This study investigated the structural, electronic, and supramolecular properties of two polymorphic forms of IBU and assessed their influence on the development and performance of emulsion-based delivery systems. The solid-state description included Hirshfeld surface analysis and the quantum theory of atoms in molecules complemented by density functional theory and electronic reactivity descriptors. Two formulations, an emulsion (EM-IBU) and a nanoemulsion (NE-IBU), were prepared and characterized by droplet size, polydispersity index, zeta potential, density, and drug content via mass spectrometry. Compared to EM-IBU, NE-IBU exhibited a considerably smaller particle size (31.3 ± 0.3 nm vs 235.4 ± 4.3 nm), a lower polydispersity index (0.23 vs 0.16), a more negative zeta potential (−25.8 vs −22.1 mV), and a higher density (0.989 vs 0.967 g cm^–3^). These quantitative results demonstrate superior colloidal stability and a wider pH stability range for NE-IBU, confirming its physicochemical robustness and pharmaceutical potential for hydrophobic drug delivery.

## Graphical abstract

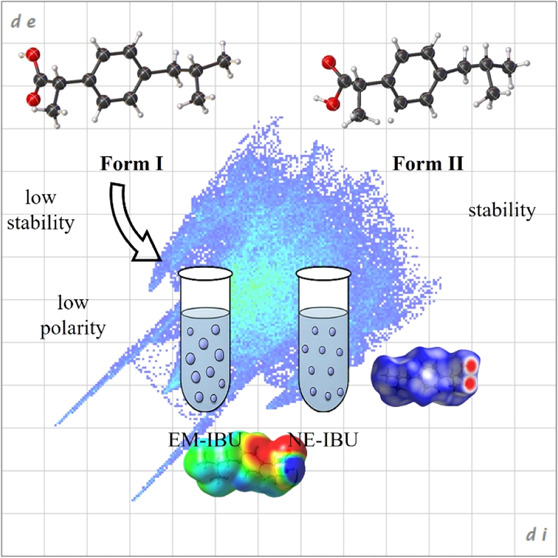

## 1 Introduction

Ibuprofen (IBU; 2-(4-isobutylphenyl)­propionic acid) is a nonsteroidal anti-inflammatory drug (NSAID) patented in 1961 by Stewart Adams and John Nicholson^1^ widely used for mild-to-moderate pain and inflammation. It is generally safe, though gastrointestinal and renal adverse effects are well documented.^2,3^ IBU is found as a racemic mixture, although only the *S*(+)-2-(4-isobutylphenyl)-propionic acid enantiomer is responsible for its pharmacological activity.^4^ However, a fraction of the *R*(−)-enantiomer can be metabolized by the 2-arylpropionyl-CoA epimerase, converting it into the biologically active *S*(+) form. Pharmacologically, IBU acts as a competitive, reversible inhibitor of COX-1 and COX-2, blocking arachidonic acid access to the catalytic site and thereby reducing the formation of prostaglandins and thromboxane A_2_.^4,5^ Its hydrophobicity and solid-state polymorphism can limit dissolution and bioavailability.^4^ Among the known crystalline forms, polymorph I, characterized on a centrosymmetric monoclinic space group *P*2_1_/*c*, is notable for its superior pharmacokinetic properties.^6^

Nanotechnology area has been widely recognized as a transformative approach for advanced pharmaceutical formulations, providing strategies to overcome solubility and targeting limitations of conventional formulations^7^ Thus, nanotechnological strategies can be employed to enhance the physicochemical properties of IBU, thereby improving its therapeutic performance. Among these, nanoemulsification stands out as a promising approach, where fine oil droplets are dispersed within an aqueous phase and stabilized by surfactants, resulting in increased solubility, bioavailability, and controlled drug release.^8^ This system offers several advantages, such as increased specific surface area, improved chemical stability, and the potential for controlled drug release.^9,10^

Molecular modeling techniques, as well as the determination of intermolecular interaction patterns in the crystalline arrangement of drugs, have proven to be important tools in understanding their behavior during the pharmacological stages of these substances, facilitating the identification and control of polymorphs during the development of nanoemulsions.^11^ Additionally, molecular modeling tools and complementary analytical techniques, such as dynamic light scattering (DLS), provide valuable insights into particle size and zeta potential.^6^ Furthermore, the influence of pH variation on nanoemulsion stability can be evaluated using automatic titrators to optimize the physicochemical parameters of nanostructured systems.^12^

In this regard, this study aimed to investigate the relationship between the structural and physicochemical properties of IBU in nanoemulsions. The electronic structure and supramolecular arrangement patterns of two distinct crystalline forms were compared, which differ in molecular conformation during crystal formation. These differences enabled a deeper understanding of the molecule’s behavior within nanostructures. By combining molecular modeling with the analysis of intermolecular interaction patterns in the supramolecular organization of IBU, this study elucidated the mechanisms underlying the efficacy and stability of pharmaceutical formulations, advancing further toward the application of nanotechnology-based techniques.^8^

## 2 Computational and Experimental Procedures

### 2.1 Solid-State Analysis

The crystallographic information file (CIF) was extracted at the Cambridge Crystallographic Data Centre (CCDC) under the codes 1179382 ^13^ (Form I) and 774097 ^14^ (Form II). IBU molecules were modeled using density functional theory (DFT),^15,16^ implemented in the Gaussian 16 software package.^17^ Theoretical calculations were performed using the highly parametrized empirical exchange–correlation functional M06-2X, accompanied by the diffuse and polarized triple-ζ basis set 6-311++G­(2d,2p). Previous studies have shown that the M06-2X functional provides accurate descriptions of medium-range electron correlation and noncovalent interactions, making it one of the most reliable choices for modeling thermodynamic properties in chemical systems.^18^ To ensure that the systems reached the ground state, frequency calculations were performed. The atomic coordinates of each molecular system were obtained from the respective crystallographic states, and relaxed scan calculations showed the most stable conformations of the IBU molecules. The geometric parameters of the molecules in the solid state were compared and then the theoretical results were compared with the experimental ones.

The supramolecular structures of Form I and Form II were compared by analyzing their intermolecular interaction patterns. To achieve this, the Hirshfeld surface (HS)^19,20^ and 2D fingerprint plots^21^ were employed to visualize and quantify these interactions. Subsequently, the interaction types were examined using quantum theory of atoms in molecules (QTAIM),^22–24^ and their stabilities were evaluated through natural bond orbital (NBO) analysis,^25^ estimated through the second-order perturbation formula

1 $$E_{i\to j^{*}}^{\left(2\right)}=-n_{\sigma }\frac{{\left\langle \sigma_{i}\left|\mathrm{F̂}\right|\sigma_{j}^{*}\right\rangle }^{2}}{\varepsilon_{j^{*}}-\varepsilon_{i}}=-n_{\sigma }\frac{F_{ij}^{2}}{\varepsilon_{j^{*}}-\varepsilon_{i}}$$

where ⟨σ|*F*|σ⟩^2^ or *F*_*ij*_^2^ is the Fock matrix element between the *i*, and *j* NBO; ε_σ*_ is the energy of the antibonding orbital σ*, and ε_σ_ is the energy of the bonding orbital σ; *n*_σ_ is the population occupation of the σ donor orbital. These combined methodologies have been successfully applied in previous crystallographic studies involving chalcone derivatives, reinforcing their applicability for analyzing intermolecular forces and electronic density in molecular crystals.^26–28^

### 2.2 Molecular Modeling

From the fully optimized structure, the energies of the Frontier molecular orbitals (FMO),^29^ the highest occupied molecular orbital (HOMO) and the lowest unoccupied molecular orbital (LUMO), were obtained. With the values of these energies in hand, the energy gap and other descriptors of chemical reactivity, namely, chemical hardness^30,31^

2 $$\eta =\frac{1}{2}{\left(\frac{\partial^{2}E}{\partial N^{2}}\right)}_{\upsilon }=\frac{I-A}{2}$$

(measuring resistance to deformation of the electron cloud during chemical processes), chemical potential^31^

3 $$\mu ={\left(\frac{\partial E}{\partial N}\right)}_{\upsilon }=-\frac{I+A}{2}=-\chi$$

(related to charge transfer from a species with higher chemical potential, μ_large_, to one with lower chemical potential, μ_small_), and global electrophilicity index^32^

4 $$\omega =\frac{\mu^{2}}{2\eta }$$

(measuring energy stabilization when the system acquires electronic charge from the environment) were obtained. The molecular electrostatic potential (MEP) map^33,34^ was obtained to locate the nucleophilic and electrophilic regions of the molecule. The electrostatic potential,^35^ *V*(**r**), at the point **r**, is defined as

5 $$V\left(r\right)=\sum_{\alpha }\frac{Z_{\alpha }}{\left|r_{\alpha }-r\right|}-\int \frac{\rho \left(r^{\prime }\right)}{\left|r^{\prime }-r\right|}\;dr^{\prime }$$

where *Z*_α_ is the charge of nuclei α at point **r**_α_ and ρ­(**r** ^′^) is the charge density at the point **r** ^′^.^36^ In addition, the chemical reactivity of IBU was compared to that of nine other NSAIDs (alminoprofen, benoxaprofen, fenoprofen, flunoxaprofen, flurbiprofen, indoprofen, ketoprofen, naproxen, and suprofen) that have the same general structure (Figure 1). The Fukui indices^37^ indicated sites where the molecule is susceptible to nucleophilic, electrophilic, and radical attacks.

6 $$f^{+}={\left[\frac{\partial \rho \left(r\right)}{\partial N}\right]}_{\upsilon }^{+}$$

7 $$f^{-}={\left[\frac{\partial \rho \left(r\right)}{\partial N}\right]}_{\upsilon }^{-}$$

8 $$f^{0}={\left[\frac{\partial \rho \left(r\right)}{\partial N}\right]}_{\upsilon }^{0}$$

**Figure 1:**
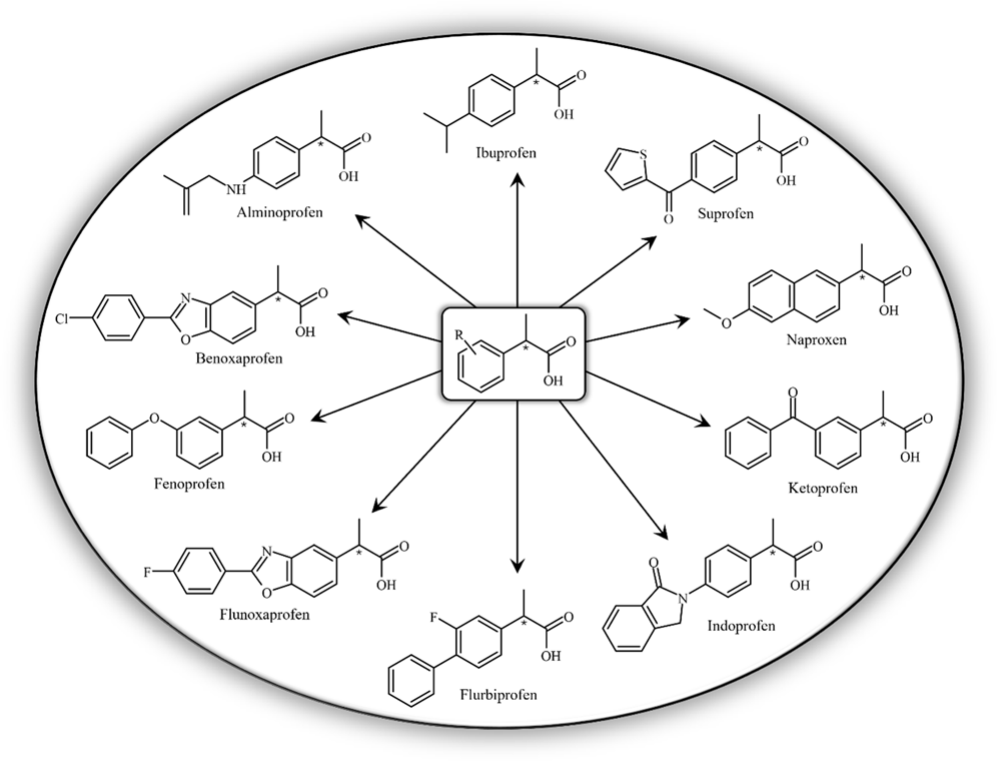
Representative structures of nonsteroidal anti-inflammatory drugs (NSAIDs) from the arylpropionic acid class, including ibuprofen and structurally related compounds. These molecules share a characteristic arylpropionic acid scaffold, which influences their physicochemical properties, molecular reactivity, and pharmacological activity.

In Equations 5, 6, 7 and 8, ρ­(**r**) is the electronic density, *N* is the electronic population, and υ is the external potential.

### 2.3 Nanotechnology Characterization

XRPD analysis was performed using a Bruker D2 PHASER diffractometer at room temperature (22.1 °C). The experimental conditions were as follows: scan range 5.00°–120.00° 2θ, step size 0.02016°, 1.0 s per step, incident beam slit 0.36°, Soller slit 2.5°, and Ni filter (2.5 μm). The analysis followed the United States Pharmacopeia (USP–NF 2024), The diffraction pattern was compared with ICDD standards, confirming Form I.^39^

IBU (1.2% w/v) was dissolved in mineral oil at 65 °C, while polysorbate 80 and purified water at the same temperature formed the aqueous phase. The oil phase was gradually incorporated into the aqueous phase under constant stirring (600 rpm) to create a coarse emulsion. This was further processed via ultrasonic homogenization (20 kHz, 40% pulse, 4 min) to achieve a nanoscale droplet size and enhance formulation stability, as described by Anuar et al.^11^ and Rai et al.^39^ The density of EM-IBU and NE-IBU was measured using a calibrated glass pycnometer 5 mL at 25 °C, calculated with the formula ρ = *m*/*V* (mass/volume). The analysis was performed in triplicate, with results expressed as mean ± standard deviation. The mass difference between the empty pycnometer and the pycnometer with the sample determined the density. The volume corresponds to the liquid phase in the formulation, and the formula used is

9 $$\rho =\frac{m_{sample}-m_{empty\;pycnometer}}{V_{pycnometer}}$$

Analyses were performed in triplicate, with results expressed as mean ± standard deviation.

The droplet size, polydispersity index (PDI), and zeta potential of EM-IBU and NE-IBU were measured using dynamic light scattering (DLS) with the Zetasizer Advance Series Pro (Malvern Instruments) at 25 °C. The Zeta potential was determined through electrophoretic mobility with samples diluted in purified water (1:100, v/v). The stability of EM-IBU and NE-IBU was evaluated using the Zetasizer Advance Series Pro coupled with an MPT-3 autotitrator. Samples were diluted in purified water (1:100, v/v) and subjected to pH adjustments (2–12) to determine the optimal colloidal stability range (±30 mV). Formulations were stored in amber glass bottles at room temperature for further analysis.

IBU content in EM-IBU and NE-IBU was quantified using mass spectrometry (MS) with electrospray ionization (ESI) in negative mode. The [M – H]^−^ ion was detected at *m*/*z* 161.1 u/e, with a retention time of 3.4 min. Samples diluted in methanol (1:1000, v/v). Independent two-sample *t* tests were applied for group comparisons, while one-way ANOVA followed by Bonferroni’s posthoc test evaluated pH titration stability across ranges. Significance levels were classified as (*p* < 0.001), (*p* < 0.01), (*p* < 0.05), and ns (*p* ≥ 0.05). Results were expressed as mean ± standard deviation (SD), and analyses were performed using Python’s scipy.stats module, with graphical data visualization including error bars to illustrate variability.

## 3 Results and Discussion

### 3.1 Solid-State Analysis

The IBU crystals analyzed are conformational polymorphs given by the rotation of the −COOH group. Both polymorphic structures contain the *R*(−) and *S*(+) stereoisomers of the drug, interconnected within the supramolecular arrangement. The crystallographic parameters are presented in Table 1. Although both structures belong to the same space group (*P*2_1_/*c*), they differ due to the rotation of the carboxyl group within the molecule (Figure 2). From the ORTEP maps (Figure 2a.b) and the superposition of the molecular structures (Figure 2c), an RMSD of 0.0213 Å allows for the clear identification of the observed molecular rotation. The geometric parameter analysis revealed that the molecular structures in the two crystal forms do not exhibit significant differences. However, the C_2_–C_3_ bond length in Form II is 1.34 times greater than in Form I, standing out as an outlier in the scatter plot Figure 3a. Additionally, the bond angles C_10_–C_11_–C_12_ and C_12_–C_11_–C_13_ in Form II are approximately 0.81 times smaller than those in Form I Figure 3b. This discrepancy may be attributed to limitations in the resolution or refinement of the crystallographic structure of Form II rather than to the intrinsic rotation of the −COOH group. The MAPD values, calculated using the equation

10 $$MAPD=\frac{100}{n}\sum_{i=1}^{n}\left|\frac{\chi_{i}-\chi_{j}}{\chi_{j}}\right|$$

were relatively high Figure 3, yet they maintained a strong correlation (*R* ^2^ > 0.77). In eq 10, χ_*i*_ and χ_*j*_ represent the theoretical and experimental geometric parameters, respectively. The C_4_–C_2_–C_1_–O_1_ torsional angles in Forms I and II were determined to be 73.49° and −81.51°, respectively. The energy scan plot of the C_2_–C_1_ bond rotation Figure 2d indicates that Form I has a higher energy than Form II, with an energy difference of approximately 0.72 kcal/mol.

**Table 1:** Crystallographic Data and Structure Refinement for IBU Form I and Form II

crystal data	form I	form II
chemical formula	C_13_ H_18_ O_2_	C_13_ H_18_ O_2_
molecular volume	306.136	318.007
crystal system	monoclinic	monoclinic
space group	*P*2_1_/*c*	*P*2_1_/*c*
temperature (K)	283–303	258
*Z*, *Z*′	4; 1	4; 1
*a* (Å)	14.667	12.379(<1)
*b* (Å)	7.886	5.872(<1)
*c* (Å)	10.730	17.562(1)
*A* (deg)	90	90
*B* (deg)	99.36	94.87(<1)
Γ (deg)	90	90
*V* (Å^3^)	1224.544	1272.028
*R*[*F* ^2^ > 2(*F* ^2^)]	3.9	5.8

**Figure 2:**
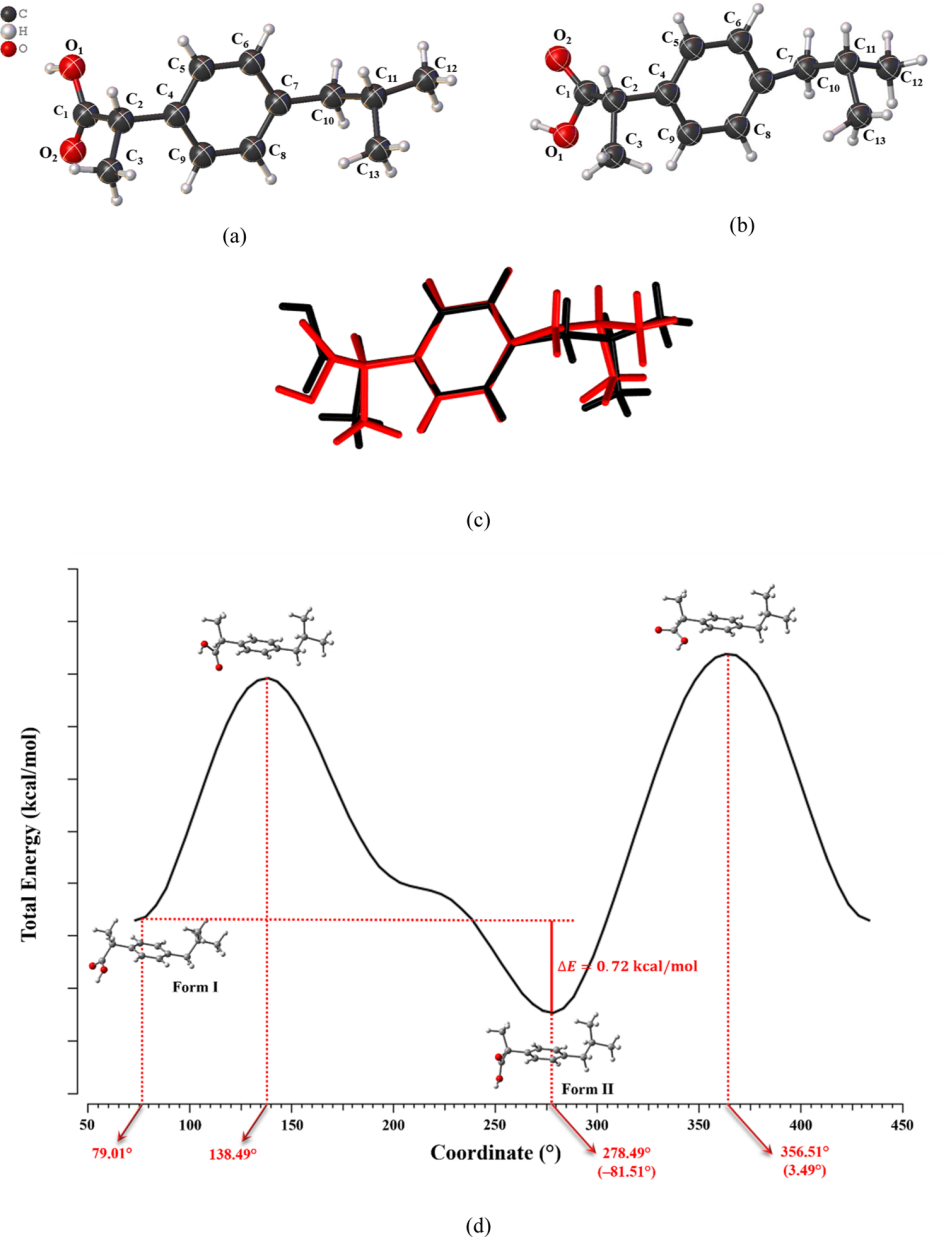
ORTEP plots of (a) Form I and (b) Form II of IBU, along with the (c) superposition of both conformers (RMSD = 0.0213 Å), where Form I is depicted in black and Form II in red. The ellipsoids are represented at a 75% probability level with the atomic numbering scheme and the hydrogen atoms are represented by spheres with arbitrary radii. The result of the relaxed scan calculation is presented in the graph (d), showing how the total energy of the molecule varies with the rotation of the C_1_–C_2_ bond.

**Figure 3:**
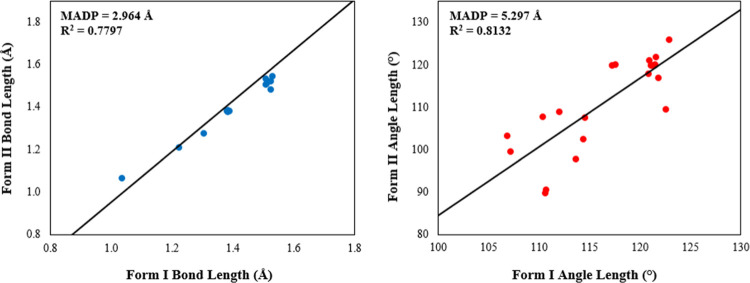
Scatterplots comparing (a) bond length and (b) bond angle in Forms I and II of IBU.

The 2D fingerprint plots reveal minor variations in the intermolecular contacts between the polymorphs, attributed to the rotation of the −COOH group. O/H and C/H contacts are more prominent in Form I, whereas H/H contacts predominate in Form II Figure 4. No C···C contacts were detected, indicating the absence of π···π stacking interactions.

**Figure 4:**
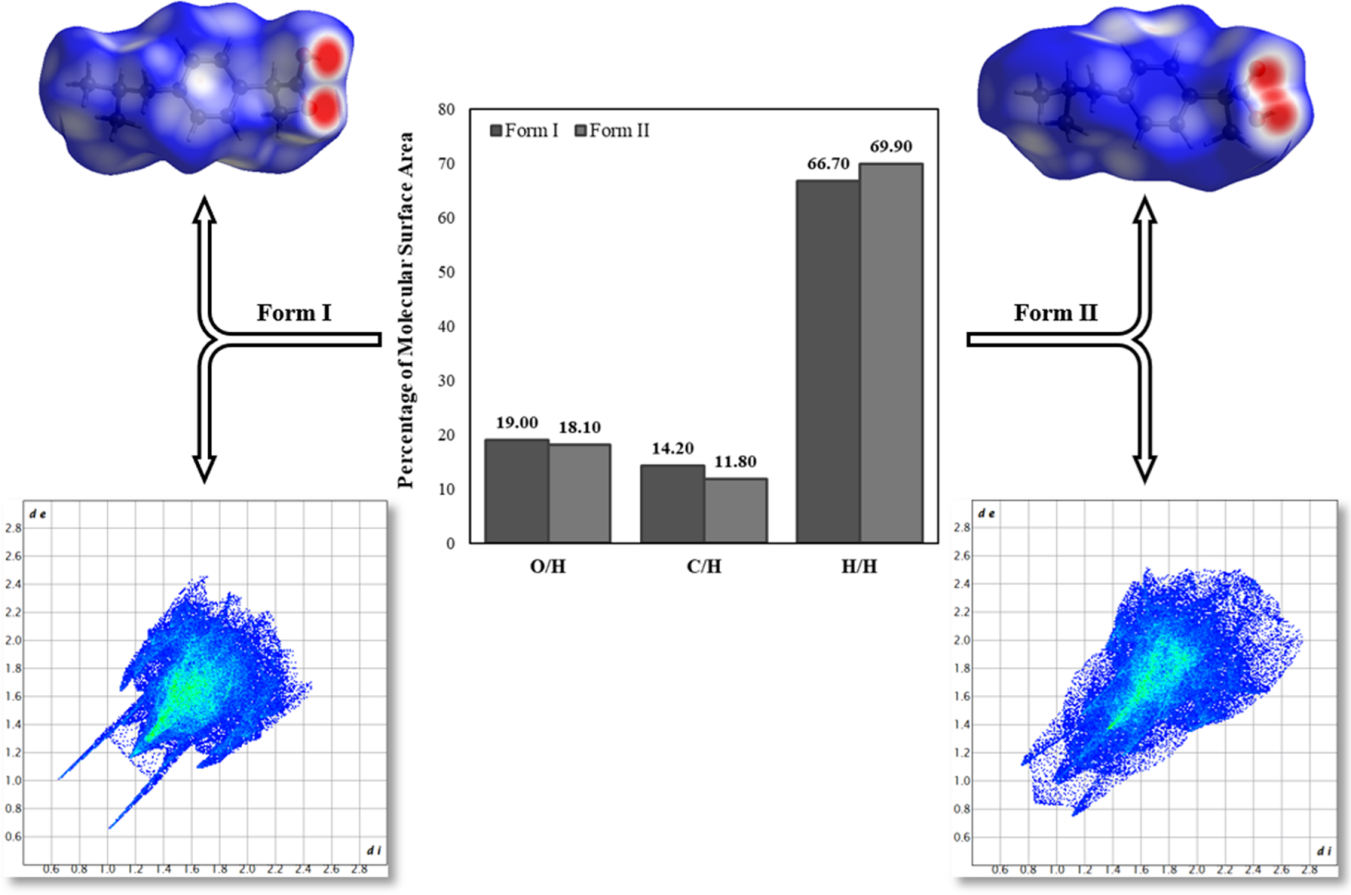
Hirshfeld surfaces (top), 2D fingerprint plots (bottom), and percentage contributions of intermolecular contacts (center) for IBU Forms I and II.

The supramolecular arrangements of both crystal forms are strongly stabilized by O_1_–H···O_2_ H-bonds, described by the graph set R_2_^2^(8),^40^ which gives rise to a common dimer (D1) in both polymorphs Figure 5. In both structures, the supramolecular ring formed by these interactions is located on the inversion center of the unit cell. Topological parameters obtained from QTAIM analysis^41^ indicate that these H-bonds exhibit ρ values of 0.0328 au in Form I and 0.0271 au in Form II at the bond critical points (BCP), with ∇^2^ρ > 0—characteristic of H-bond,^24,42^ as described by Nakanishi.^43,44^ Molecular systems containing interactions of this order resulted in similar topological parameters.^45–47^ Table 2 summarizes the QTAIM topological data for the supramolecular arrangements of the two forms.

**Figure 5:**
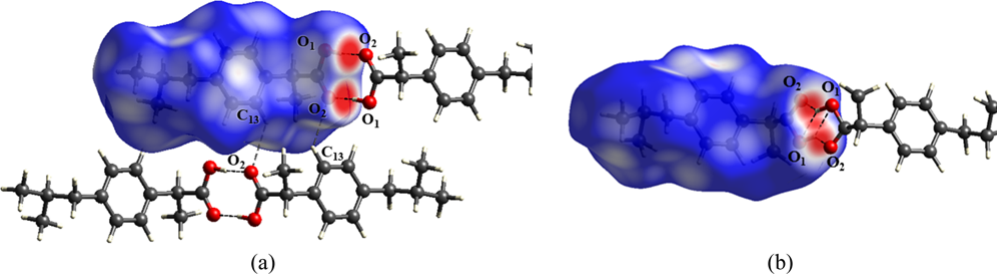
Hirshfeld surfaces and dimers formed by the main intermolecular interactions in the crystal structures of IBU Forms (a) I and (b) II.

**Table 2:** Topological Parameters from QTAIM Analysis of the Intermolecular Interactions in the Crystallographic Forms of IBU, Obtained at the M06-2*X*/6-311++G­(d,p) Level of Theory

interaction	ρ (a.u.)	∇^2^ρ (a.u.)	***G*** (**r**) (a.u.)	***v*** (**r**) (a.u.)	***h*** (**r**) (a.u.)	$\frac{\left|v\right|}{G}$	interaction type
Form I							
O_1_–H···O_2_	0.03282	0.26022	0.06100	–0.05694	0.00406	0.9	H-bond
O_1_–H···O_2_	0.03281	0.26016	0.06098	–0.05693	0.00406	0.9	H-bond
C_12_–H···O_2_	0.00460	0.01957	0.00409	–0.00329	0.00080	0.8	van der Waals
C_13_–H···O_2_	0.00080	0.02768	0.00594	–0.00495	0.00098	0.8	van der Waals
C_13_–H···H–C_13_	0.00175	0.00706	0.00137	–0.00098	0.00039	0.7	van der Waals
C_6_–H···C_11_	0.00546	0.01435	0.00304	–0.00250	0.00055	0.3	van der Waals
C_12_–H···H–C_7_	0.00167	0.00716	0.00140	–0.00102	0.00039	0.7	van der Waals
Form II							
O_1_–H···O_2_	0.02711	0.15962	0.03773	–0.03556	0.00217	0.9	H-bond
O_1_–H···O_2_	0.01202	0.08643	0.01890	–0.01619	0.00271	0.9	H-bond
O_1_–H···O_2_	0.02711	0.15960	0.03773	–0.03556	0.00217	0.9	H-bond
C_11_–H···H–C_3_	0.00701	0.02348	0.00486	–0.00384	0.00101	0.8	van der Waals
C_2_–H···C_8_	0.00448	0.01814	0.00393	–0.00333	0.00060	0.8	van der Waals
C_2_–H···C_9_	0.00627	0.02046	0.00420	–0.00329	0.00091	0.8	van der Waals
C_6_–H···H–C_12_	0.00466	0.01599	0.00334	–0.00269	0.00065	0.8	van der Waals

The higher ρ observed in Form I, combined with the shorter O_1_–H···O_2_ distance (1.62 Å), corresponds to a binding energy (BE) of −21.97 kcal/mol, calculated using the counterpoise method to correct for basis set superposition error (BSSE).^48^ In contrast, Form II exhibits an 11.1% longer H-bond distance and a markedly weaker BE of −3.21 kcal/mol. These findings indicate that the hydrogen bond in dimer D1 is significantly stronger in Form I than in Form II. Orbital analysis shows that the H-bond is primarily stabilized by interactions between the σ­(O_1_–H) and π­(O_2_) orbitals, with greater contributions in Form I (51.26% and 22.97%, respectively) compared to Form II (49.29% and 19.84%). This difference is associated with the larger O_1_–H–O_2_ bond angle in Form I (174.6°), which favors a more linear and cohesive geometry. In contrast, the angular distortion in Form II (138.9°) diminishes the interaction strength.

In Form I, a second dimer (D2) is also observed, stabilized by C_13_–H···O_2_ interactions and described by the graph set R_2_^2^(12). According to QTAIM, this interaction is classified as a van der Waals-type, with ρ = 0.007 au and ∇^2^ρ > 0. The associated BE is estimated at −4.76 kcal/mol, representing a secondary but relevant contribution to the crystal cohesion. Two D1 units are connected through D2 Figure 5a, forming a motif that propagates along the *b*-axis, with H bonds aligned in the *ab* plane. As a result, the crystal structure of Form I consists of alternating layers, giving rise to distinct polar and nonpolar regions.

In Form II, the D1 units are connected through weak H···H contacts, with an estimated interaction energy of −0.17 kcal/mol. No additional dimers were identified in this crystal form. Nonetheless, the structure also organizes into discrete polar and nonpolar regions, although with lower intermolecular cohesion compared to Form I. Beyond structural and energetic distinctions, the existence of multiple crystalline forms of IBU also raises important regulatory and biopharmaceutical considerations. Polymorphism can influence not only solubility and dissolution rates but also affect the stability, manufacturability, and bioavailability of solid pharmaceutical products.^49^ Moreover, variations in polymorphic forms can compromise batch-to-batch consistency and therapeutic equivalence, potentially impacting the approval process of generic formulations.^50^ Thus, understanding the supramolecular organization and intermolecular interaction strength of IBU polymorphs is not only scientifically relevant but also essential for ensuring regulatory compliance and clinical efficacy.

The supramolecular arrangements of the IBU polymorphs reveal significant differences that can critically impact their physicochemical and pharmacotechnical properties. The presence of stronger and topologically cohesive hydrogen bonds—characterized by higher ρ values and more linear bond angles—correlates with a more stable, densely packed, and less soluble crystalline structure.^51^ Such features may contribute to enhanced physical stability and improved behavior during storage and industrial processing. In contrast, weaker intermolecular interactions, geometric distortions, and lower binding energies are associated with increased solubility and faster dissolution rates—properties that are particularly desirable in formulations designed for rapid drug release.^50^ Furthermore, the supramolecular organization of the crystal lattice, characterized by alternating polar and nonpolar domains stabilized through hydrophobic and van der Waals interactions, directly influences crystal morphology, compaction, and powder flowability, ultimately affecting drug bioavailability. Therefore, the selection of the appropriate polymorph for pharmaceutical formulations of IBU must carefully balance solid-state stability, solubility, and therapeutic efficacy, according to the intended profile of the final product.

### 3.2 Molecular Modeling Analysis

The isosurfaces of the FMOs of IBU stereoisomers are shown in Figure 6, and no significant energetic differences were observed between the isomers. The HOMO is a π orbital, whereas the LUMO exhibits diffuse character (Rydberg orbital caracter), which accounts for its high energy and low electron density. The *R*(−) isomer was found to be slightly more stable. However, data from the scientific literature indicate that *S*(+) is the pharmacologically active enantiomer, responsible for the antipyretic and anti-inflammatory effects. The *R*(−) isomer, in turn, acts as a partial prodrug, with approximately 60% of its structure being converted into *S*(+) in vivo, thereby prolonging the drug’s therapeutic response.^52^ The greater electronic stability of *R*(−) observed in the calculations can be attributed to its lower chemical reactivity, reflected by the slightly wider energy gap (Δ*E*_H‑L_). This difference does not imply greater biological activity but rather an energetically more stable electronic configuration. In a biological context, the relative stability of *R*(−) is offset by its metabolic conversion to *S*(+), which exhibits superior stereochemical compatibility with the active site of cyclooxygenase (COX). Thus, the theoretical and experimental results are complementary: *R*(−) is slightly more electronically stable, whereas *S*(+) is more pharmacodynamically efficient due to its stronger molecular affinity for the enzymatic target.^53^

**Figure 6:**
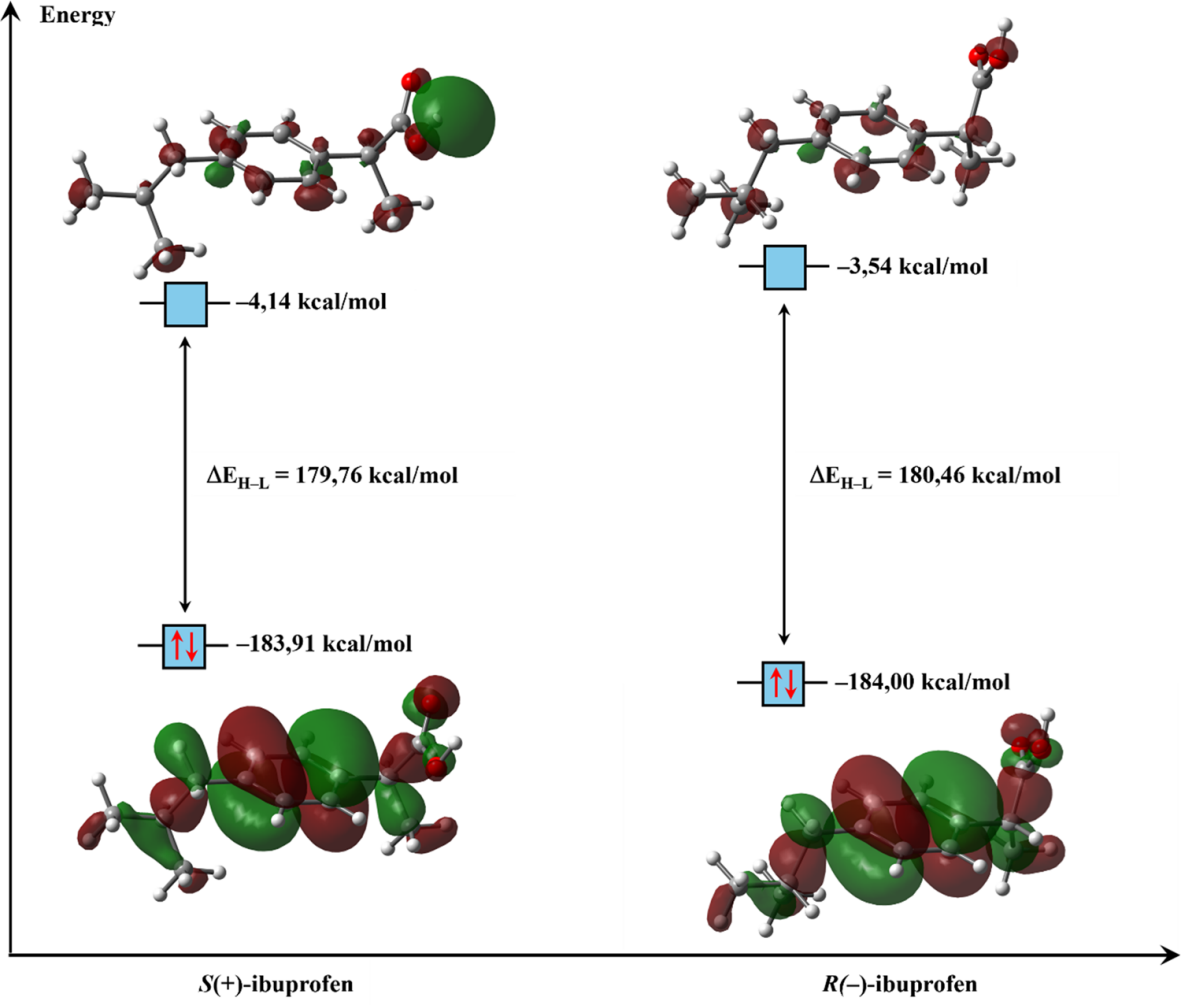
Isosurfaces of the Frontier molecular orbitals of ibuprofen stereisomers, HOMO and LUMO, (isovalue = 0.02 au), illustrating electron density distribution, obtained at M06-2*X*/6-311++G­(2d,2p) level of theory.

Comparing the *S*(+) stereoisomer to other NSAIDs, the IBU molecule exhibits intermediate basicity compared to other drugs in the same chemical group. The *E*_H_ values range from −160.45 kcal/mol (for AMP) to −194.34 kcal/mol (for KTP), with IBU being approximately 14.6% less fundamental than AMP and 5.4% more basic than KTP (Table 3). The high basicity of AMP is attributed to its amine group. Additionally, the high ionization energy of IBU (183.91 kcal/mol) indicates that the drug is a weak reducing agent. Since the LUMO of IBU is a Rydberg orbital, the compound has the highest *E*_L_ value, which limits its orbital stability and prevents it from being the most acidic in this group. Furthermore, its low electron affinity (4.15 kcal/mol) suggests that IBU is not highly susceptible to accepting electrons from nucleophilic species. The *E*_L_ values range from −4.15 kcal/mol (IBU) to −29.52 kcal/mol (SUP), indicating that IBU is the least acidic drug in this group, whereas SUP is the most acidic. The higher acidity of SUP is associated with the moderately electronegative thiophene ring, which acts as an electron-withdrawing group, facilitating the deprotonation of the acidic hydrogen atom of the −COOH group.

**Table 3:** Chemical Reactivity Descriptors of Ibuprofen and Other NSAIDs Obtained at the M06-2*X*/6-311++G­(2d,2p) Level of Theory^a^

descriptor	IBP	AMP	BNP	FNP	FLP	FBP	INP	KTP	NPX	SUP
HOMO energy, *E*_H_	–183.91	–160.45	–177.05	–170.82	–176.56	–179.89	–171.40	–194.34	–162.04	–192.05
LUMO energy, *E*_L_	–4.15	–4.25	–26.46	–4.59	–22.44	–10.97	–16.75	–23.52	–13.24	–29.52
energy gap, Δ*E*_H‑L_ ^b^	179.76	156.20	150.59	166.23	154.13	168.91	154.65	170.81	148.80	162.53
ionization energy, *I*	183.91	160.45	177.05	170.82	176.56	179.89	171.40	194.34	162.04	192.05
electronic affinity, *A*	4.15	4.25	26.46	4.59	22.44	10.97	16.75	23.52	13.24	29.52
electronegativity, χ	94.03	82.35	101.76	87.70	99.50	95.43	94.07	108.93	87.64	110.78
chemical potential, μ	–94.03	–82.35	–101.76	–87.70	–99.50	–95.43	–94.07	–108.93	–87.64	–110.78
chemical hardness, η	89.88	78.10	75.29	83.11	77.06	84.46	77.32	85.41	74.40	81.26
electrophilicity index, ω	49.18	43.42	68.76	46.27	64.24	53.92	57.23	69.46	51.62	75.51

a Descriptor values are reported in kcal/mol.

b Δ*E*_H‑L_ = *E*_L_ – *E*_H_.

The energy gap, ΔE_H‑L_, and chemical hardness values suggest that IBU is the most electronically stable compound in this group. The presence of the Rydberg orbital in the IBU molecule influences its chemical reactivity, making it less prone to reactions with nucleophiles, in addition to contributing to overall chemical stability. Similar quantum chemical approaches have been successfully applied to the reactivity analysis of natural bioactive compounds, reinforcing the relevance of these descriptors in drug design studies.^46,54,55^

Biologically, the high LUMO energy may result in the prolongation of the integrity of the drug molecule before oxidative metabolism. Stable drugs are less prone to undergoing unwanted metabolic transformations or generating reactive intermediates. Moreover, the stability of IBU can prevent interactions with reactive biomolecules, reducing the likelihood of causing collateral damage to proteins and nucleic acids in cellular structures. In contrast, NPX is the most reactive compound in this group, being approximately 20.8% more reactive than IBU. This increased reactivity allows NPX to interact more effectively with COX-2, enhancing its potency by enabling faster responses to the biological target.

Furthermore, NPX is the most reactive compound in this group, being approximately 20.8% more reactive than IBU. This increased reactivity enables NPX to interact more effectively with COX-2, enhancing its potency and prolonging its duration of action. In contrast, IBU exhibits higher chemical stability but a shorter elimination half-life (1.2–2 h) compared to NPX (12–17 h). These pharmacokinetic differences indicate that IBU is more suitable for acute pain relief requiring rapid onset and short duration, whereas NPX is better suited for long-term management of chronic inflammatory conditions due to its sustained plasma levels.

Among the NSAIDs of the propionic acid group analyzed, AMP exhibits the lowest global electrophilicity index, while SUP shows the highest. Comparatively, IBU is 13.27% more electrophilic than AMP and 34.9% less electrophilic than SUP, placing it among the compounds with the lowest reactivity toward nucleophiles. According to the findings of Domingo–Pérez et al.,^56,57^ IBU can be classified as a strong electrophile, capable of participating in nucleophilic addition reactions. Furthermore, the IBU molecule displays the lowest molecular polarity index (MPI) among the NSAIDs, with a value of 8.58 kcal/mol. This value is 62.8% lower than that of water and 5.32% higher than that of benzene, indicating a tendency toward nonpolarity Figure 7a.

**Figure 7:**
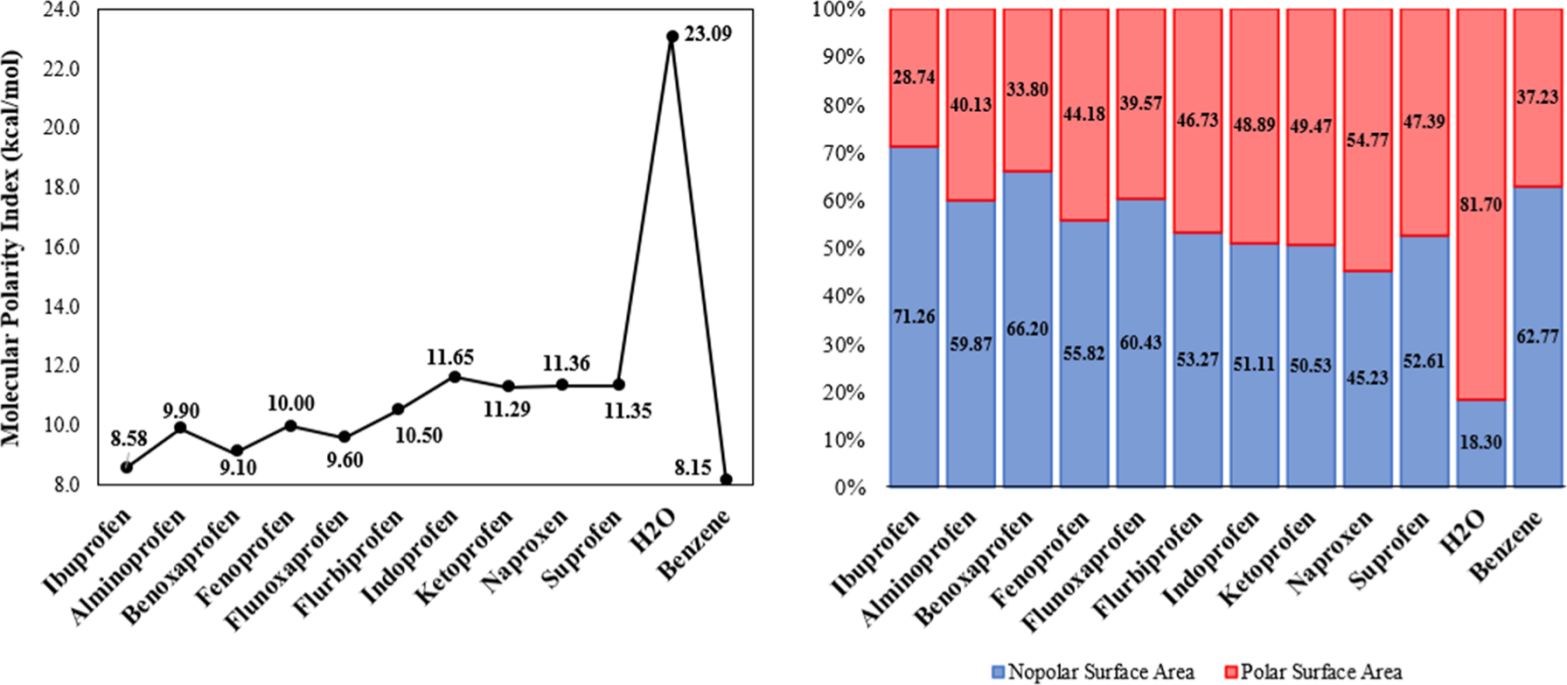
Variation in the polarity of NSAIDs from the arylpropionic acid group. (a) Molecular polarity index (MPI) values and (b) polar and nonpolar surface area contributions of these drugs, both compared to those of water and benzene.

In contrast, INP has the highest MPI at 11.65 kcal/mol. A higher MPI value indicates a greater degree of polarity. Only 28.74% of the IBU molecule’s surface area is polar; by comparison, water and benzene molecules have polar surface areas of 81.70% and 37.23%, respectively Figure 7b. The INP molecule has a polar surface area of 48.89%. All other NSAIDs in this group have more polar molecules than IBU, which can be attributed to the presence of more electronegative atoms along their carbon chains. Among them, the NPX molecule is the most polar, with a polar surface area of 54.77%. Greater polarity is associated with more clearly defined nucleophilic and electrophilic regions on the MEP surface. These features help explain IBU’s low solubility in water (21.0 mg/L at 25C) and its moderate lipophilicity (log *P* ranging from 2.48 to 3.50). The MEP map of IBU indicates that the most polarized region is located near the carboxyl group, with the carbonyl oxygen atom presenting the highest electron density (*V*(**r**) = −34.633 kcal/mol). In contrast, the acidic hydrogen atom shows the lowest electron density (*V*(**r**) = +51.020 kcal/mol).

### 3.3 Ibuprofen Nanoemulsion System

XRPD experimentally confirmed the solid-state phase (Figure 8). The diffractogram showed well-defined peaks with angular positions (2θ) and interplanar spacings (*d*) consistent with the reference pattern PDF 00-034-1728 from the International Centre for Diffraction Data (ICDD), corresponding to polymorph I of IBU.^13,38,58,59^ The most intense reflections were observed at 2θ values of 6.01°, 16.50°, 20.01°, and 22.22°, with relative intensities of 100%, 92.41%, 87.20%, and 97.97%, respectively. The sample exhibited a crystallinity of 88.4%, and no additional polymorphic phases were detected. The observed diffraction pattern matched the unit cell parameters described for polymorph I (*a* = 14.85 Å, *b* = 7.885 Å, *c* = 10.785 Å; α = 90.00°, β = 99.36° and γ = 90.00°; space group *P*2_1_/*c*), as previously reported in the literature.^59,60^

**Figure 8:**
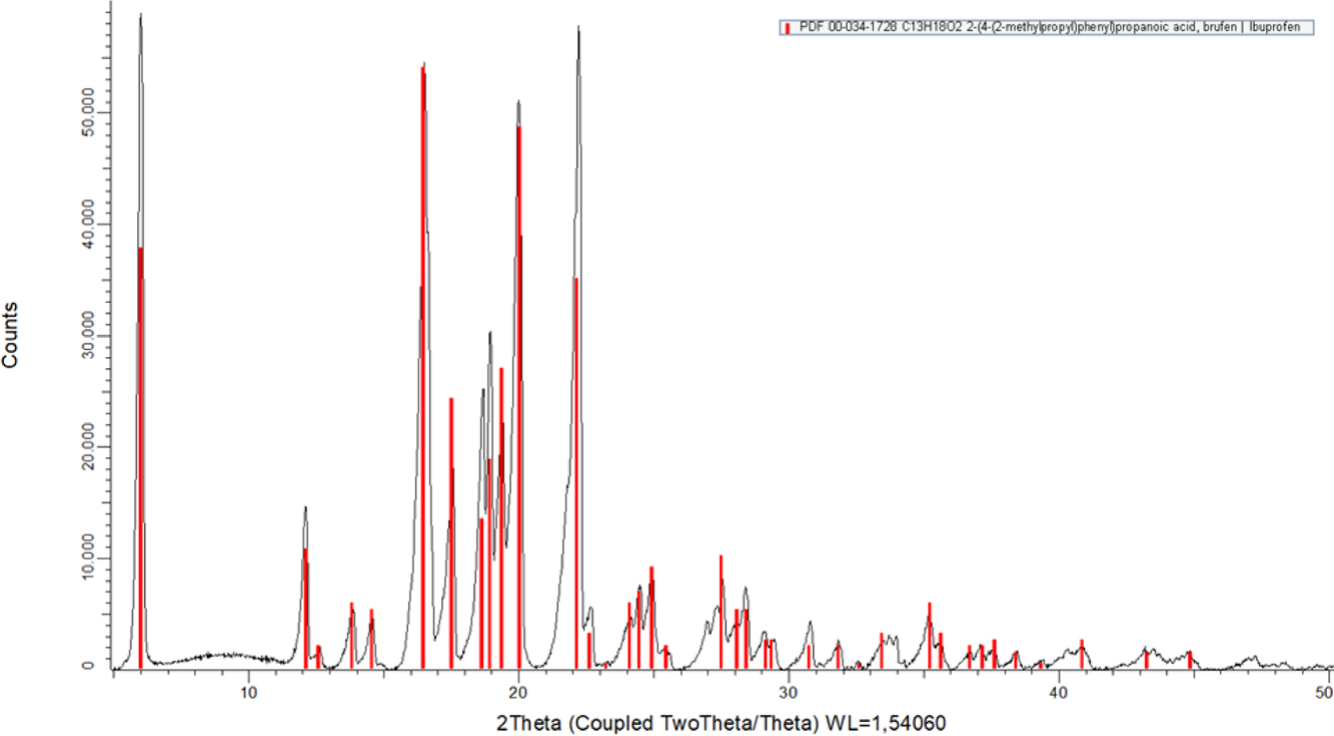
Superimposed X-ray powder diffraction (XRPD) pattern of IBU sample (black line) and the reference diffraction pattern of Ibuprofen polymorph I (red bars), from the ICDD database [PDF 00-034-1728, C_13_H_18_O_2_]. The close match between experimental and reference peaks confirms the crystalline structure as polymorph I, with high phase purity.

The complete experimental diffractogram is shown in Figure 8, and the key diffraction parameters—2θ values, interplanar distances (*d*), and relative intensities—are summarized in Table S1 (Supporting Information). These results confirm that the IBU analyzed was in its most thermodynamically stable crystalline form, reinforcing its suitability for pharmaceutical applications. Studies have demonstrated that nanoconfined environments in emulsified systems may influence the crystallization behavior and polymorphic transitions of hydrophobic drugs, including IBU, potentially altering their dissolution and bioavailability profiles.^61^

The nanoemulsion (NE-IBU) displayed significantly smaller particle sizes (31.32 ± 0.29 nm) compared to the emulsion (EM-IBU, 235.40 ± 4.25 nm, *p* < 0.001) on day 1. The size reduction achieved through ultrasonic homogenization highlights its efficiency in producing nanometric droplets, critical for increasing the surface area and enhancing drug absorption. After 30 days at pH 8.0, NE-IBU maintained its size (32.20 ± 0.29 nm, ns), indicating stability over time. EM-IBU also remained stable (234.77 ± 3.89 nm, ns), but its larger droplet size limits its potential for advanced pharmaceutical applications, where nanoscale dimensions are preferred for bioavailability and efficacy. Additional physicochemical data—including polydispersity index (PDI), zeta potential, and density—are summarized in Table 4, highlighting the stability and performance differences between the two formulations over time.

**Table 4:** Physicochemical Parameters of Ibuprofen Nanoemulsion (NE-IBU) and Emulsion (EM-IBU) Formulations Measured on Day 1 and Day 30^a^

formulation	day	size (nm)	PDI	zeta potential (mV)	density (g/cm^3^)
NE-IBU	1	31.32 ± 0.29	0.23 ± 0.00	–11.85 ± 0.64	0.989 ± 0.012
NE-IBU	30	32.20 ± 0.29	0.13 ± 0.02 (***)	–25.80 ± 1.48 (***)	—
EM-IBU	1	235.40 ± 4.25	0.16 ± 0.01	–17.44 ± 0.75	0.967 ± 0.002
EM-IBU	30	234.77 ± 3.89	0.16 ± 0.01 (ns)	–22.14 ± 1.24 (ns)	—

a Data are presented as mean ± standard deviation (*n* = 3). (***) Statistically significant difference compared to Day 1 (*p* < 0.01). (ns) Not statistically significant (*p* > 0.05).

The progressive increase in the magnitude of the negative zeta potential observed for NE-IBU (−11.85 ± 0.64 mV to −25.80 ± 1.48 mV) over 30 days can be attributed to the slow rearrangement and adsorption of polysorbate 80 molecules at the oil–water interface during nanoemulsion maturation. This interfacial reorganization enhances surface-charge density and electrostatic repulsion between droplets, reducing aggregation and improving colloidal stability. Although EM-IBU has shown minor variations over time, its larger droplet size (∼235 nm) and broader particle size distribution limit its stability. From a manufacturing perspective, the improved electrostatic stabilization and reduced PDI of NE-IBU after storage indicate superior long-term stability desirable for industrial formulation processes.

From a pharmacological perspective, the nanoemulsified form of ibuprofen may offer significant advantages in terms of bioavailability, onset of action, and protection against physicochemical degradation. The enhanced stability observed suggests that nanoemulsions can preserve the structural integrity of ibuprofen during storage and administration, reducing the formation of degradation byproducts that may compromise efficacy or safety. Additionally, the small droplet size and surfactant composition characteristic of nanoemulsions can improve mucosal permeability and gastrointestinal absorption, potentially allowing for lower doses to achieve therapeutic plasma concentrations. This may result in a reduced frequency of adverse effects, such as gastrointestinal irritation, which are dose-dependent in nonsteroidal anti-inflammatory drugs (NSAIDs). Therefore, the formulation of ibuprofen into a nanoemulsion not only enhances its physicochemical resilience but also aligns with pharmacological goals of optimizing therapeutic index, patient compliance, and formulation safety.

The density of NE-IBU (0.989 ± 0.012 g/cm^3^) was significantly higher than EM-IBU (0.967 ± 0.002 g/cm^3^, *p* < 0.001), attributed to the more compact and homogeneous structure of NE-IBU achieved through ultrasonic processing. This increased density suggests improved formulation consistency, critical for precise drug dosing. FTIR analysis confirmed the chemical integrity of IBU in both formulations, as no significant shifts or changes were observed in characteristic bands. This indicates that the preparation process did not alter the drug’s chemical structure, ensuring its therapeutic potential is preserved.

NE-IBU demonstrated improved particle uniformity over time, with PDI decreasing from 0.23 ± 0.00 on day 1 to 0.13 ± 0.02 after 30 days (*p* = 0.004), reflecting system maturation and stabilization. EM-IBU maintained a consistent but higher PDI (0.16 ± 0.01 ns), suggesting less uniform particle distribution, which could affect its reproducibility in pharmaceutical applications. Zeta potential analysis revealed superior electrostatic stability for NE-IBU, with values improving from −11.85 ± 0.64 mV on day 1 to −25.80 ± 1.48 mV after 30 days at pH 8 (*p* < 0.01). In contrast, EM-IBU showed moderately negative zeta potential values that changed from −17.44 ± 0.75 mV to −22.14 ± 1.24 mV (*p* < 0.01), Figure 9. The stronger negative zeta potential in NE-IBU reduces particle aggregation and ensures long-term colloidal stability, making it more suitable for advanced drug delivery systems.

**Figure 9:**
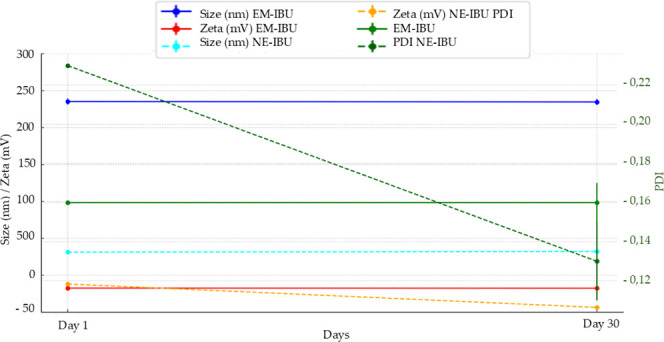
Particle size, polydispersity index (PDI), and zeta potential of EM-IBU and NE-IBU formulations measured on Day 1 and after 30 days. NE-IBU showed a significant reduction in PDI (*p* = 0.004) and an increase in negative zeta potential (*p* < 0.01), indicating improved colloidal stability and particle uniformity. EM-IBU remained relatively stable over time. Data are expressed as mean ± standard deviation (*n* = 3).

Titration revealed that NE-IBU is stable over a broader pH range (6.5–10.0), maintaining zeta potential consistently below −25 mV (ns). EM-IBU exhibited a narrower stability range (7.5–11.0), with zeta potential between −20 and −23 mV (ns). This broader stability range of NE-IBU aligns with physiological conditions such as the small intestine (pH ∼ 6.5–7.5) and skin (pH ∼ 5.5), making it a versatile candidate for both oral and topical applications, Figure 10.

**Figure 10:**
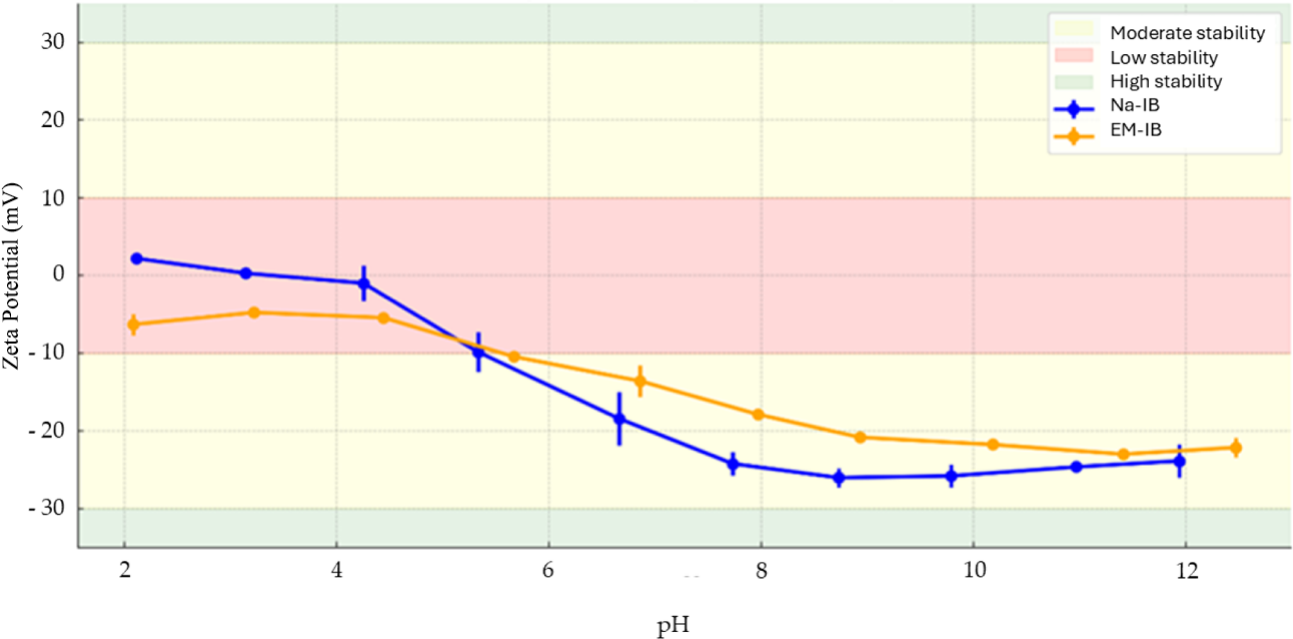
Zeta potential of NE-IBU and EM-IBU formulations as a function of pH. The shaded regions represent colloidal stability classifications: green (|zeta| > 25 mV = high stability), yellow (|zeta| ≈ 15–25 mV = moderate stability), and red (|zeta| < 15 mV = low stability). NE-IBU exhibited high stability across a broader pH range (6.5–10.0) compared to EM-IBU (7.5–11.0), indicating better performance under physiological pH conditions relevant to oral and topical drug delivery. Data are expressed as mean ± standard deviation (*n* = 3).

The quantification of IBU in NE-IBU revealed a concentration of 6.93 mg/mL, significantly lower than that observed in EM-IBU (12.23 mg/mL, *p* < 0.05). This reduction can be attributed to insufficiency of surfactant during the ultrasonic process, a phenomenon widely reported in the literature.^11^ Gupta et al. (2016)^62^ demonstrated that inadequate surfactant concentrations produce unstable droplets and reduced encapsulation efficiency. Similar effects in emulsified systems undergoing ultrasonication. These findings corroborate the results of the present study, since the larger interfacial area generated by ultrasonication, combined with the limited availability of surfactant, restricts droplet stabilization and consequently reduces drug entrapment. Future studies will focus on optimizing surfactant proportion and processing parameters to maximize encapsulation efficiency and long-term stability of NE-IBU.

The possibility of thermal or mechanical degradation of the drug was ruled out, considering the high boiling point of IBU (157–160 °C). Despite the reduction in IBU content, the encapsulated amount in NE-IBU remained within therapeutic limits for clinical applications, such as 1–5% for topical use or adjusted doses for intestinal release in oral administration. The preservation of key properties, such as physicochemical stability, nanometric particle size, and zeta potential, demonstrates that the efficiency of the nanoformulation was maintained, ensuring its functionality for the delivery of hydrophobic drugs like IBU. The nanoemulsification strategy employed in this study proved highly effective for encapsulating IBU, a hydrophobic and poorly water-soluble drug, while preserving its essential physicochemical properties. The results confirmed that the nanostructured formulation not only enabled efficient dispersion of the active compound in the aqueous phase but also provided colloidal stability and potential for application in advanced drug delivery systems. Other nanocarrier systems reported in the literature have also demonstrated effectiveness in encapsulating hydrophobic drugs, resulting in improved aqueous solubility, stability, sustained release, and increased bioavailability.^63,64^

These results reinforce the potential of nanoformulations in overcoming topical delivery challenges associated with lipophilic compounds. The enhanced delivery and stability achieved with NE-IBU parallels the benefits seen in nanoparticle and scaffold systems as highlighted in previous reviews.^7^ Solid-state analysis and molecular modeling provided critical insights into the behavior of IBU in nanoemulsion systems. The identification of conformational polymorphism and the characterization of supramolecular interactions—particularly the strength and geometry of hydrogen bonds—were essential for understanding the structural stability of the drug and its potential interactions with formulation excipients. Furthermore, the analysis of the electronic structure offered predictive information regarding the solubility profile and chemical reactivity of IBU, supporting its classification as a moderately lipophilic and weakly polar compound.

The use of nanostructured carriers to enhance drug dispersion and bioavailability, as nanoemulsions can overcome the solubility limitations of hydrophobic drugs by increasing interfacial surface area and maintaining the drug in a kinetically stabilized dispersed phase. Similar strategies have been reported for other poorly water-soluble drugs, where the combination of crystallographic and theoretical approaches has led to improved formulation outcomes.^4,8,9^ Therefore, the physicochemical rationale derived from these analyses not only validates the formulation strategy adopted but also reinforces the importance of computational and structural methodologies in the rational design of advanced drug delivery systems.

## 4 Conclusion

The findings of this study demonstrate that NE-IBU offers substantial advantages over EM-IBU in terms of physicochemical characteristics, colloidal stability, and pharmaceutical applicability. The reduced particle size, lower polydispersity index, and more negative zeta potential observed in NE-IBU formulations directly contribute to enhanced dissolution rates, improved bioavailability, and prolonged shelf stability under physiological pH conditions. These properties are further supported by solid-state and quantum chemical analyses, which reveal that the supramolecular organization and lower reactivity of IBU contribute to its compatibility with nanoformulation systems. In particular, the strong and cohesive hydrogen bonding network in Form I correlates with enhanced structural integrity, while the moderate electrophilicity and low polarity of IBU align with its hydrophobic nature and demand for solubilization strategies. Collectively, these results highlight the synergistic value of combining crystallographic, computational, and nanotechnological approaches in the design of optimized drug delivery nanosystems. NE-IBU thus represents a promising nanoplatform for delivering poorly water-soluble drugs, ensuring both therapeutic efficacy and formulation robustness.

## Supplementary Material



## Notes

Crystallographic data for the ibuprofen sample used in the preparation of the nanoemulsion system are provided in the Supporting Information. The principal diffraction parameters—2θ values, interplanar spacings (*d*), and relative intensities—are summarized in Table S1. These results confirmed that the analyzed ibuprofen corresponded to its most thermodynamically stable crystalline phase, supporting its suitability for pharmaceutical formulation. This structural characterization served as the basis for the subsequent computational and nanotechnological analyses reported in this study. Angular position (2θ), interplanar spacing (*d*), and relative intensity (%) obtained from the X-ray powder diffraction analysis of the Ibuprofen sample IBP (PDF)
