# Supplementary material for: Integrating Molecular Modeling and Nanoemulsion Characterization for Ibuprofen
Source: ACS Omega. 2026 Feb 25;11(9):14456–68. doi: 10.1021/acsomega.5c09579 (PMC12980217; doi:10.1021/acsomega.5c09579)
Supplement: Supplementary file 1 [file ao5c09579_si_001.pdf]

Integrating Molecular Modeling and Nanoemulsion Characterization for Ibuprofen

Antônio S. N. Aguiar[1,2], Luana A. F. Afiune[1,3], Vitória A. M. Silva[1], Rodrigo A. B. Lopes-Martins[4], Lucas D. Dias[1], Alberto S. S. Filho[1], James O. Fajemiroye[1,5], Leonardo L. Borges[2,6], Hamilton B. Napolitano[1,2]

[1]Laboratório de Novos Materiais, Universidade Evangélica de Goiás, Anápolis, GO, Brazil, 75083-515.
[2]Grupo de Química Teórica e Estrutural de Anápolis, Universidade Estadual de Goiás, Anápolis, GO, Brazil, 75132-400.
[3]Laboratório de Pesquisas em Biodiversidade, Universidade Evangélica de Goiás, Anápolis, GO, Brazil, 75083-515.
[4]Centro Universitário UniRedentor, Itaperuna, RJ, Brazil, CEP 28300-000.
[5]Instituto de Ciências Biológicas, Universidade Federal de Goiás, Goiânia, GO, Brazil, 74690-900
[6]Escola de Ciências Médicas e da Vida, Pontifícia Universidade Católica de Goiás, Goiânia, GO, Brazil, 74605-010.

Supporting Information

The key diffraction parameters – 2θ values, interplanar distances (d), and relative intensities – are summarized in Table S1. These results confirm that the Ibuprofen analyzed was in its most thermodynamically stable crystalline form, reinforcing its suitability for pharmaceutical applications. This structural profile was used as the basis for subsequent computational and nanotechnological analyses conducted in this study.

Table S1. Angular position (2θ), interplanar spacing (d), and relative intensity (%) obtained from the X-ray powder diffraction analysis of the Ibuprofen sample IBP.

No.	Angular position (2θ)	Interplanar spacing (Å)	Relative intensity (%)
1	6,014 (4)	14,68468	100,00
2	12,107 (6)	7,30427	25,01
3	13,856 (7)	6,38611	9,47
4	14,559 (6)	6,07925	8,93
5	16,506 (5)	5,36621	92,41
6	17,565 (7)	5,04498	30,57
7	18,668 (6)	4,74935	42,90
8	19,369 (6)	4,57789	42,21
9	20,012 (6)	4,43333	87,20
10	22,218 (4)	3,99793	97,97
11	22,663 (6)	3,92033	9,73
12	24,125 (7)	3,68602	7,99
13	24,478 (6)	3,63364	12,76
14	24,919 (7)	3,57028	14,94
15	27,328 (6)	3,26084	9,85
16	25,517 (7)	3,23885	13,90

17	28,097 (6)	3,17329	8,30
18	28,375 (5)	3,14289	12,65
19	29,093 (6)	3,06686	6,02
20	29,434 (8)	3,03215	4,77
21	30,796 (5)	2,90109	7,47
22	31,842 (6)	2,80808	4,77
23	33,469 (8)	2,67523	4,87
24	33,721 (6)	2,65579	5,39
25	33,986 (8)	2,63570	5,08
26	35,209 (7)	2,54689	9,02
27	35,613 (8)	2,51895	4,56
28	36,686 (6)	2,44767	3,27
29	37,107 (5)	2,42089	3,92
30	37,586 (7)	2,39110	3,31
31	38,453 (6)	2,33920	2,91
32	40,303 (8)	2,23597	3,11
33	40,858 (7)	2,20686	3,72
34	43,188 (6)	2,09306	3,56
35	43,499 (7)	2,07880	3,23
36	44,761 (6)	2,02309	2,67
